# Primary Headache Attributed to External Compression or Traction to the Head: A Narrative Review

**DOI:** 10.1002/brb3.70202

**Published:** 2024-12-30

**Authors:** Ole Hensel, Torsten Kraya

**Affiliations:** ^1^ University Clinic and Outpatient Clinic for Neurology Martin‐Luther‐University Halle‐Wittenberg Halle/Saale Germany; ^2^ University Clinic and Outpatient Clinic for Radiology Martin‐Luther‐University Halle‐Wittenberg Halle/Saale Germany; ^3^ Department of Neurology St. Georg Hospital Leipzig Germany

**Keywords:** external‐compression headache, external‐traction headache, helmet headache, hijab headache, ponytail headache

## Abstract

**Background:**

The aim of this review is to synthesize the existing knowledge regarding headaches attributed to external physical stimuli, as classified by the ICHD‐3 (Group 4.6). Two forms can be distinguished in this group: (1) headache attributed to external compression and (2) headache attributed to external traction.

**Methods:**

A comprehensive literature review was conducted using the Medline (PubMed) database and other relevant academic sources. All English‐language articles were subjected to a relevance assessment.

**Results:**

The prevalence of the two types of headache varies considerably, with a higher incidence observed in women or in the presence of predisposing factors (e.g., work with compulsory helmets or long hair). An external‐compression headache is typically described as a pressing sensation, whereas an external‐traction headache is characterized by a sensation of pulling. The headaches typically persist for less than an hour after the stimulus has ceased, and the intensity is typically reported as mild to moderate. Apart from avoiding the trigger, which is not always possible, effective therapies have not been established.

**Conclusion:**

Both types of headaches are relatively common, yet they remain underrepresented in the scientific literature. Furthermore, there is a dearth of comprehensive overviews. If the triggering factor cannot be eliminated, both types of headaches can interfere with daily living and working.

## Graphical abstract

Headache attributed to external compression and headache attributed to external traction are relatively common, yet they remain underrepresented in the scientific literature. If the triggering factor cannot be eliminated, both types of headaches can interfere with daily living and working.

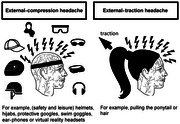

**Abbreviation**
VAS: Visual Analog Scale

## 1 Introduction

Headache is a common symptom with numerous causes, many of which are well documented. Among the lesser‐known forms of primary headache are compression headache and traction headache in the ICHD‐3 under the items IHS 4.6.1 and 4.6.2 (ICHD‐3). Both types of headaches lead to an impairment of quality of life if the triggering factor cannot be eliminated. In certain instances, those affected are forced to change jobs due to the unavailability of prophylactic measures for safety reasons, coupled with the absence of a suitable treatment. The external‐compression headache (Table 1) and external‐traction headache (Table 2) are relatively infrequently reported, often underdiagnosed, and rarely reported scientifically. As a result, physicians may not be very aware of these specific types of headaches. Raising awareness of these two types of headaches among general practitioners, neurologists, and researchers is therefore necessary. Understanding their etiology, pathophysiology, and clinical features can help to better recognize this type of headache and avoid confusing it with other, more common types of headache. Informing patients about these rare headache types can improve their quality of life, as accurate diagnosis, treatment, and prophylaxis can help to relieve headaches and prevent recurrences. The aim is to identify research gaps and focus attention on these poorly understood headache types. Overall, the review article on external‐compression headache and traction headache could not only contribute to the medical literature but also improve clinical practice, enhance patient quality of life, and stimulate new research initiatives.

**TABLE 1:** Summary of the external‐compression headache based on ICHD 4.6.1 (International Headache Society 2018).

Previously used terms or synonyms	Headache due to external compression, swim‐goggle headache, football‐helmet headache, and hijab (headscarf) headache.
Triggers	Eye or virtual reality goggles, tight hats, professional and recreational helmets, headbands, hairbands, hijabs, hearing protection, in‐ear and over‐ear headphones, wigs, and others.
Description	Headache resulting from sustained compression of pericranial soft tissues, e.g., by a tight band around the head, hat or helmet, or goggles worn during swimming or diving, without damage to the scalp.
Diagnostic criteria	At least two episodes of headache fulfilling criteria B–D, Brought on by and occurring within 1 h during sustained external compression of the forehead or scalp, Maximal at the site of external compression, Resolving within 1 h after external compression is relieved, and Not better accounted for by another ICHD‐3 diagnosis.

**TABLE 2:** Summary of the external‐traction headache based on ICHD 4.6.1 (International Headache Society, 2018).

Previously used terms or synonyms	Headache due to external traction, ponytail headache, and “hair‐triggered migraine”
Triggers	Hair extensions, braid/ponytail, heavy hair, jewelry, or pulling the hair
Description	Headache resulting from sustained traction on pericranial soft tissues, without damage to the scalp.
Diagnostic criteria according to ICHD 4.6.1	At least two episodes of headache fulfilling criteria B–D Brought on by and occurring only during sustained external traction on the scalp, Maximal at the traction site, Resolving within 1 h after traction is relieved, and Not better accounted for by another ICHD‐3 diagnosis.

## 2 Methods

A search for this narrative review was conducted in the peer‐reviewed abstracts in Medline (PubMed) and in other academic, partly non–peer‐reviewed full texts in the Consensus.ai (based on the Semantic Scholar database) and the Google Scholar database. The search terms “external compression headache” “external‐compression headache,” “headache due to external compression,” “swim‐goggle headache,” “football‐helmet headache,” “hijab headache” and “external traction headache,” “external‐traction headache,” “headache due to external‐traction,” “ponytail headache,” and “hair‐triggered migraine” were employed in all three databases. The last check was done on July 12, 2024. We found 81 items of varying relevance during the review process; they were subjected to a review by O.H. and T.K. to ascertain their relevance with respect to epidemiology, clinical presentation, pathophysiology, therapeutic modalities, and associations with other headache disorders. For comparison, since 2020, there have been over 100 articles in PubMed that contain the terms “covid‐19,” “personal protective equipment,” and “headache” in the abstract. Articles pertaining to mask‐induced headaches or COVID‐19 and personal protective equipment were excluded from the review. An analysis of this literature would go beyond the scope of the review. Further restrictions were not imposed (e.g., age of the subjects and publication date) due to the limited number of findings.

## 3 External‐Compression Headache (ICHD 4.6.1)

The International Classification of Headache Disorders (ICHD) has classified headache due to external compression as a distinct category within the broader category of headaches caused by external pressure (International Headache Society, 2018), a classification that has been in place since the first edition of the ICHD. It is imperative to differentiate between primary and secondary headaches, particularly in instances where external factors are involved.

### 3.1 Epidemiology

A survey of adults (25‐ to 64‐year‐old) stated that the lifetime prevalence of external‐compression headaches is 4% (Rasmussen and Olesen 1992). External‐compression headache was reported by only 0.02% of people who visited a specialized tertiary headache center (Lupi et al. 2018). Most sufferers consult their general practitioner first. The prevalence is higher in regions where headgear is prevalent or mandatory for cultural or religious reasons. One example is wearing the hijab in Islamic countries: all hijab wearers interviewed reported headaches, which they attributed to wearing the hijab (Ansari and Solomon 2015). Wearing a hijab or headscarf has a deeper meaning in Islam and is seen as a sign of faith or belonging. There are many more differences between cultures in how often hats are worn (Weiss, Kirsner, and Hu 2012). It has been observed that individuals engaged in specific occupational activities, such as construction work or aviation, who frequently utilize tight headgear, such as helmets or headphones, respectively, may demonstrate elevated rates of this condition (Rahmani et al. 2017; Krymchantowski et al. 2004). It is a legal requirement to wear a hard hat on construction sites, in mining, in many factory buildings, or when using certain industrial vehicles. This is to avoid head injuries from falling objects. Approximately 12% of motorcyclists surveyed reported that they had already developed an external‐compression headache while riding with a motorcycle helmet (Ahmad et al. 2022). As the causes of an external‐compression headache are readily identifiable, and the pain subsides when the compression is removed, individuals experiencing these symptoms often refrain from seeking medical attention. Consequently, the actual prevalence of these incidents may be higher than reported. Among those who wore helmets, 30% of the study participants reported experiencing headaches (Rahmani et al. 2017). Military doctors have confirmed that external‐compression headaches are common among female military personnel (Franklin, Wohltmann, and Won 2021). The occurrence of external‐compression headaches is higher in women as well (Rasmussen and Olesen 1992). This may be attributed to the higher rates of this type of headache among women. Additionally, an increased prevalence of external‐compression headaches was also observed in males with migraine (Krymchantowski et al. 2004). The occurrence of external‐compression headaches is also contingent upon the characteristics of the head covering in question (Rahmani et al. 2017). When cultural, religious, or safety concerns make it difficult to detach the headgear causing the headache, prolonged irritation may result and explain why external‐compression headache is more common in specific groups (e.g., military personnel, motorcyclists, or hijab wearers).

### 3.2 Clinic

External‐compression headaches have been observed to occur predominantly within the initial hour following continuous or repeated mechanical stimulation (Krymchantowski 2010; Fernández‐Garza and Marfil 2020). Examples of potential triggers include helmets, hijabs, (protective or swim) goggles, earphones, or virtual reality headsets.

The most frequently reported pain type was a “pressing pain,” with “throbbing‐pulsating” and “stabbing‐sharp” being less prevalent (Ansari and Solomon 2015; Rahmani et al. 2017; Krymchantowski et al. 2004; Krymchantowski 2010).

The most severe pain was localized in the area where pressure was applied (Rahmani et al. 2017; Krymchantowski 2010). If the headgear is distributed evenly, as is the case with helmet wearers, for example, the headache is more diffusely distributed (Krymchantowski 2010; Fernández‐Garza and Marfil 2020). The majority of reported headaches were located in the frontal region of the head (Rahmani et al. 2017).

The intensity of the headache was moderate, with an average rating of 4 on the Visual Analog Scale (VAS), ranging from 0 to 10 (Rahmani et al. 2017; Krymchantowski et al. 2004; Krymchantowski 2010). In 55% of respondents, attributed external‐compression headaches had a substantial impact on their daily lives. A woman reported experiencing an external‐compression headache on a daily basis when she wore the hijab (Ansari and Solomon 2015).

The duration of time over which an external compression must act in order to trigger an external‐compression headache has yet to be systematically investigated. The time required for nociceptor stimulation to provoke a headache in an external‐compression headache is likely to vary between individuals and is dependent on a number of factors, including the individual's pain threshold, the intensity of the applied pressure, and the duration of the compression. However, patients report that sustained nociceptor activation generally takes longer than a few seconds or minutes to result in the onset of a headache and also that the intensity of the headaches increases with duration.

The majority of subjects (Ansari and Solomon 2015; Krymchantowski et al. 2004) reported that the headaches had disappeared after the trigger was removed, with the vast majority of cases resolving within an hour. In a small number of individuals, however, the headache can persist for up to 5 days (Fernández‐Garza and Marfil 2020). In each seventh individual, the sustained use of the helmet resulted in the onset of a pulsating, more intense, unilateral headache accompanied by nausea and photophobia (Krymchantowski et al. 2004). Migraine attacks may also be precipitated (Rasmussen and Olesen 1992; Franklin, Wohltmann, and Won 2021). Additionally, external‐compression headaches can manifest with a number of secondary effects. It is a significant contributing factor to the observed reluctance among children to wear helmets when riding bicycles (Ahmad et al. 2022). In times of war, military personnel were compelled to be replaced due to the onset of helmet‐related headaches (Pierce, Palombaro, and Black 2014).

As external‐compression headaches are frequently precipitated by the use of helmets, they are often classified as occupational diseases. It is therefore recommended that external‐compression headache be taken into account in the development of protective helmets designed for professional use (Cohen et al. 2012). It is of the utmost importance to ensure that individuals susceptible to external‐compression headaches are not compelled to wear such protective helmets, for example, due to the heightened risk of accidents that such a choice may entail. The level of discomfort or comfort associated with virtual headsets can have a rapid and significant impact on the sales of commercial products.

A comparison with the clinic of external‐traction headache is shown in Table 3.

**TABLE 3:** Comparison of the clinical characteristics of external‐compression headache and external‐traction headache.

	External‐compression headache (Ansari and Solomon 2015; Rahmani et al. 2017; Krymchantowski et al. 2004; Ahmad et al. 2022; Franklin et al. 2021; Krymchantowski 2010; Fernández‐Garza and Marfil 2020)	External‐traction headache (Barreto et al. 2020; Blau 2004; Abbas et al. 2022)
Incidence	4%, higher in women and headgear wearers	40%–54%, higher in woman, people with long hair, and higher age
Location	place of irritation, mostly forehead, temples, back of the head	directly to the pigtail, parietal, frontal, vertex and neck, bilateral
Pain type	pressing, (throbbing‐pulsating, stabbing‐sharp)	pulling, pressing, non‐pulsatile
Duration of irritation before headache start	minutes to max. 60 min	16 s to max. 60 min
Duration of headache after end of irritation	minutes to few days	15 s to 13 h (mean 76 min) after loss of the trigger
Onset time	seconds to minutes	
Intensity of headache	4 of 10 on VAS	mild to moderate, rarely severe
Exemplary triggers	tight hats, hijabs, helmets, glasses, headbands, earphones, virtual reality headsets	hair extensions, braid/ponytail, heavy hair, jewelry, or pulling the hair
Relieving factors	removing or adjusting the pressure source, breaks	removing the traction source, tying the hair 1 cm from the scalp, massaging painful areas
Association to other primary headaches	in migraine patients: triggering of a pulsating, more severe, unilateral headache associated with nausea and photophobia	In migraine patients: rarely a migraine‐like episode are triggered, increased headache intensity during physical exertion; primary headache precondition or a migraine disorder increases the likelihood

Abbreviation: VAS, Visual Analog Scale.

### 3.3 Pathophysiology

**FIGURE 1:**
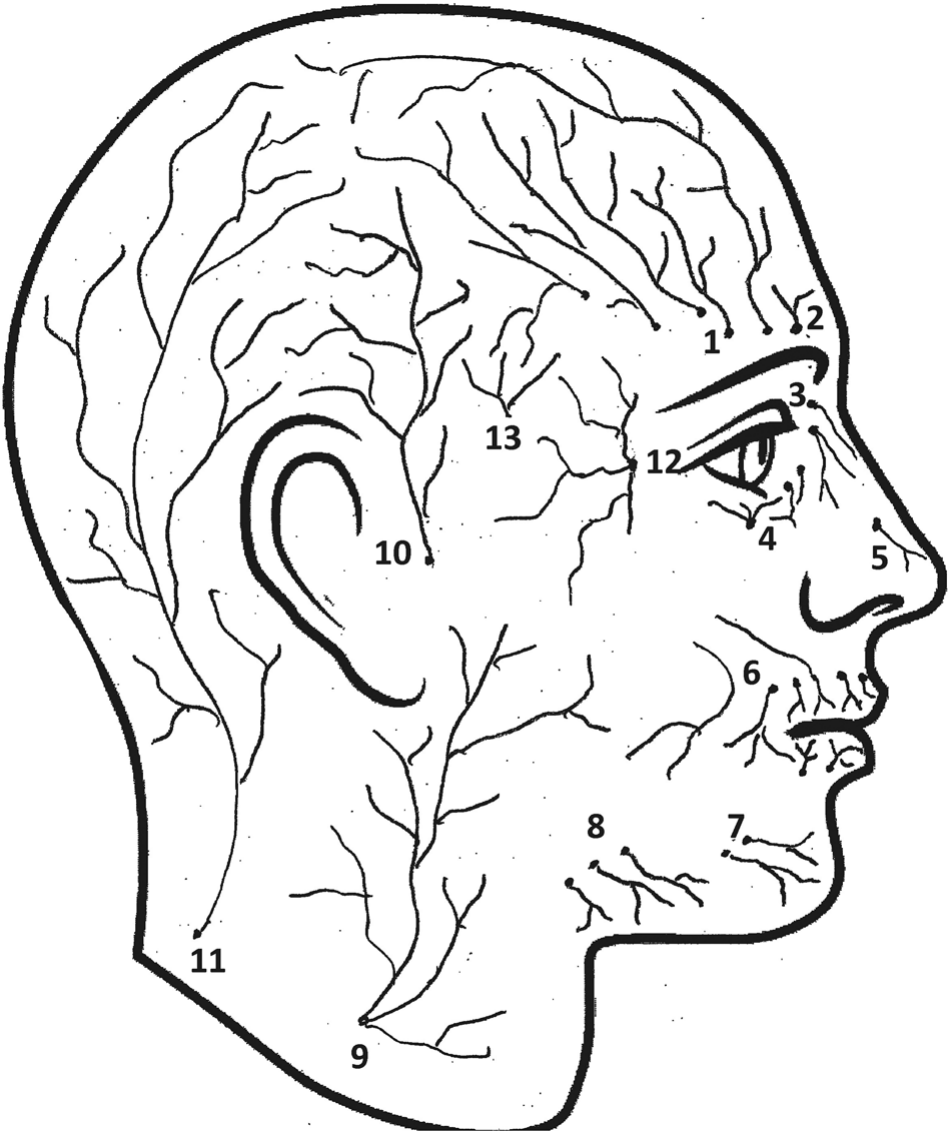
Superficial cutaneous (sensory) nerves of the face and the scalp. (1) Supraorbital nerve (lateral and medial), (2) supratrochlear nerve, (3) infratrochlear nerve, (4) palpebral branches of infraorbital nerve, (5) anterior ethmoidal nasal nerve, (6) labial and mental branches of infraorbital nerves, (7) labial and mental nerves, (8) buccal nerve, (9) great auricular nerve, (10) auriculotemporal nerve, (11) greater occipital nerve, (12) zygomaticofacial nerve, and (13) zygomaticotemporal nerve.

It is hypothesized that external‐compression headaches activate superficial cutaneous nerves of the face and the scalp (Figure 1) by sustained external, compressive forces on pericranial structures (Krymchantowski 2010; Fernández‐Garza and Marfil 2020). The pericranial structures are composed of the following elements: the skin, subcutaneous tissue, epicranial muscles enclosed by the fascia covering them, subaponeurotic loose areolar tissue, and the pericranium. This would be analogous to neuralgia, a pain state that occurs without evidence of tissue damage. Functional connections traverse the skull, establishing interconnections between extracranial and intracranial nerves. Mechanical stimulation or irritation of extracranial tissues (e.g., from the periosteum or muscles) or nociceptors co‐activates meningeal pain fibers, which exert a direct influence on the development of headaches (Burstein et al. 2017; Schueler et al. 2013, 2014, Schueler et al. 2013, 2014).

However, this does not elucidate why some individuals develop external‐compression headaches, whereas others do not. In order for external, nonstructural, damaging pressure to cause a headache, additional factors must be taken into consideration.

Given that cutaneous nociceptors require prolonged stimulation before external‐compression headaches manifest, it is plausible that this prolonged stimulation may result in central or peripheral sensitization, thereby increasing the excitability of central neurons and ultimately leading to the headache. It is also possible that sensitization may activate the trigeminocervical complex. This hypothesis was tested in a patient who was required to wear a hard hat at work and subsequently developed a drug‐resistant external‐compression headache. The patient's headache remitted after a 7‐month period of abstinence from wearing a hard hat (Sadamoto 2023). It is similarly plausible that diffuse noxious inhibitory control is diminished. This has been demonstrated in the context of tension headaches (Bezov et al. 2011).

A deeper comprehension of the mechanisms underlying nociceptor stimulation may facilitate the development of more efficacious preventive and therapeutic strategies for the management of external‐compression headaches. For example, helmets, headphones, or virtual reality headsets could be designed in a manner that precludes the triggering of external‐compression headaches. However, external‐compression headache still needs to be better understood, for instance, in order to develop better tolerable headgear. We found no studies in which this type of headache was experimentally induced. Further research is needed in this direction.

### 3.4 Therapy and Prophylactic Measures

When general practitioners or neurologists are consulted about external‐compression headache, the focus is on advices about the cause, treatment, and prevention. The resolution or prevention of external‐compression headaches can be achieved by the avoidance of the precipitating cause. In the event that an external‐compression headache has been caused by headgear, it may be possible to alter the types or sizes of the headgear in question. It has been demonstrated that regular breaks with loosening or removal of the tape will reduce the incidence of headaches from external compression (Krymchantowski et al. 2004). Nevertheless, this approach may not be effective for all individuals with a history of migraine headaches (Krymchantowski et al. 2004). One woman who habitually wore a hijab was compelled to abstain from it for a period of 2 years, during which time the frequency and intensity of her symptoms diminished (Ansari and Solomon 2015). If safety helmets or other protection hats must be used for different reasons, and this results in an external‐compression headache, the employee is legally entitled to claim this as an occupational disease.

It is beneficial to provide education about the benign nature of these conditions and to make educational materials available. The design of protective helmets, such as the contact surface, better cushioning, and weight, has been demonstrated to impact the incidence of external‐compression headaches (Rahmani et al. 2017). The use of helmets with special inner padding, which exert uniform pressure on multiple regions of the head, has been demonstrated to result in a reduction in the frequency of external‐compression headaches (Rahmani et al. 2017). In the event that wearing a protective helmet is unavoidable, it may be advisable to consider a prophylactic regimen of pain medication, such as naproxen with a relatively long half‐life. Conversely, individuals who continued to utilize their helmets reported that the analgesic medications, which typically alleviated their typical headaches, were ineffective in relieving their external‐compression headaches (Krymchantowski et al. 2004).

Table 4 summarizes practical recommendations for the prevention of external‐compression headache.

**TABLE 4:** Practical strategies based on our experience to avoid external‐compression headache.

*Avoidance of the precipitating cause*: If possible, remove the triggering factor.
*Loosen the band*: If possible, adjust the tightness of the helmet or hijab. A looser fit can reduce pressure on the head.
*Padding*: Use padding or cushioning inside the helmet or around the hijab to distribute pressure more evenly.
*Try other models*: Try other models of headwear. Different padding will prevent headaches.
*Scheduled breaks*: If the situation allows, take regular breaks to remove the helmet or hijab and relieve pressure.
*Gentle massage*: During breaks, gently massage the scalp and areas where the helmet or hijab applies pressure.
*Stay hydrated*: Drink plenty of water, as dehydration can exacerbate headaches.
*Balanced diet*: Ensure regular meals and snacks to maintain stable blood sugar levels, which can also help in headache prevention.
*Posture awareness*: Maintain good posture, especially if wearing a helmet, to avoid additional tension in the neck and shoulders.
*Neck exercises*: Incorporate gentle neck stretches and exercises to relieve tension.
*Cool compress*: Use a cool cloth or ice pack on the forehead or back of the neck during breaks to alleviate headache symptoms.
*Breathable fabrics*: Opt for lightweight, breathable materials for the hijab that allow for better airflow and temperature control.
*Slow adjustment*: If new to wearing a helmet or hijab, gradually increase the duration of wear to allow the body to adjust.

Our literature research found no evidence that alternative therapeutic measures (such as biofeedback, acupuncture, relaxation techniques, or aromatherapy) are effective. There is clearly a need for further research in this area.

### 3.5 Associations With Other Headache Disorders

External‐compression headaches are distinguished from other primary headache disorders by their distinctive characteristics, triggers, and underlying mechanisms. However, external‐compression headaches were markedly more prevalent among individuals with migraine than in those without migraine (Rasmussen and Olesen 1992). Additionally, Krymchantowski et al. (2004) observed that among a predominantly male cohort of police officers, 13% of all participants and 31% of those with migraine reported a headache associated with wearing a helmet. Moreover, 44% of individuals with external‐compression headaches reported a history of intermittent headache attacks with characteristics suggestive of migraine, and 21% reported previous headache attacks with features suggestive of tension‐type headaches (Krymchantowski et al. 2004). The palpation of certain points on the scalp triggers a migraine attack in those affected (Calandre et al. 2006).

It is not clear whether there are common pathophysiological mechanisms between headaches caused by external compression and migraine. It is also possible that external‐compression headache triggers the migraine. The pathophysiology of each may be different, but both groups of headaches may have a lowered threshold for pain recognition.

In other cases, a preexisting primary headache disorder may increase the likelihood of an external‐compression headache, the intensity of the pain, and the duration of the attack. Additionally, there are therapeutic interactions. For example, the application of a rubber band around the head has been demonstrated to provide pain relief for migraines (Vijayan 1993).

People with migraines or other primary headache disorders should avoid wearing accessories on their head, as these could trigger or exacerbate headaches.

### 3.6 Differentiation From Mask‐Induced Headache

Since the advent of the 2020 coronavirus disease (COVID‐19) pandemic, a considerable number of scientific articles have been published examining the phenomenon of headaches induced by the use of protective masks, such as those designated as filtering facepiece type 2 (FFP2) masks. Some of the articles posit that this headache is a form of external‐compression headache (Ong et al. 2021; Jafari et al. 2021; Ong et al. 2020). From our perspective, however, there are notable distinctions beyond the apparent similarities, leading us to postulate the existence of distinct headache types. The act of wearing protective masks has been observed to elicit a series of pronounced physiological alterations. As a consequence of the reduction in temperature exchange, there is an increase in body temperature and a secondary increase in fluid excretion. The exhaled air demonstrates an elevated pCO_2_ (hypercapnia), which subsequently results in an increased respiratory rate, heart rate, and chest discomfort. This also elucidates why wearing a protective mask elevates the blood flow velocity in the middle cerebral artery by 10% (own data, not yet published). It would be possible that the so‐called hijab headache has similar effects. A reduction in oxygen concentration in bodily tissues has been observed. It is of interest to determine whether there is any influence on the development of headaches during the current pandemic, and if so, what factors may be involved. These may include existing anxiety, the particular psychological stress, increased work pressure, and secondary sleep deprivation. The combination of a face mask and a breathing mask with active air purification and additional retention was found to be more comfortable than the use of a face mask alone (Bharatendu et al. 2020). This suggests that the headache was not caused by external compression. In addition to the headache, studies have reported several other neurological complications following the use of protective masks. Of these, sleep disturbances, poor concentration, and irritability are particularly noteworthy (Ramirez‐Moreno et al. 2021). Furthermore, the use of face masks has been associated with an increased occurrence of other symptoms, including poor concentration, fatigue, dizziness, sleep disturbances, and nasal congestion. These observations have been reported in individuals experiencing a mask‐induced headache, as well as in those who do not have a headache but use masks regularly (Joy et al. 2021). These reasons indicate that a mask‐induced headache should not be classified as an external‐compression headache. Accordingly, this article does not address that particular issue.

## 4 External‐Traction Headache (ICHD 4.6.2)

### 4.1 Epidemiology

External‐traction headaches are more prevalent among individuals with long hair, particularly women. Despite the prevalence of external‐traction headaches, there is a paucity of literature on this particular type of headache. The prevalence of external‐traction headaches in females ranges from 40% to 54% (Barreto et al. 2020; Blau 2004). Additionally, external‐traction headaches were experimentally induced more frequently in the older age groups (Barreto et al. 2020).

### 4.2 Clinic

The latency to the onset of the experimental external‐traction headache ranged from 16 s to 1 h (Barreto et al. 2020). The external‐traction headaches were predominantly nonpulsatile in character. In 6% of cases of provoked external‐traction headaches, a migraine‐like episode was triggered. Photophobia or phonophobia was observed in 21% of individuals experiencing external‐traction headaches. The prevalence of external‐traction headache symptoms was found to increase with physical exertion in 31% of subjects. It is noteworthy that these observations were made exclusively in patients with a history of migraine headaches (Barreto et al. 2020). External‐traction headaches were predominantly bilateral (Barreto et al. 2020) and localized directly to the pigtail in 20% of cases (Blau 2004). In particular, the location was parietal in 16% of cases, frontal in 14%, and vertex and neck in 10% each (Blau 2004). The majority of subjects (64%) reported mild pain, whereas 31% experienced moderate pain, and only 6% reported high pain intensity (Barreto et al. 2020). External‐traction headaches have a mean duration of 76 min, with a range of 15 s to 13 h. In 64% of subjects, the headaches last less than 30 min. The headaches occur after the braid or ponytail is loosened (Barreto et al. 2020; Blau 2004; Abbas et al. 2022).

It is well established in the hair care industry that ponytails that are against the direction of hair growth are a primary cause of external‐traction headaches.

A comparison to external‐compression headache is described in Table 3.

### 4.3 Pathophysiology

It is hypothesized that the sensitive cutaneous nerves situated beneath the hair attachments may be irritated, and that the traction of extracranial muscles, fasciae, or tendons may also be a potential cause of the observed pain (Barreto et al. 2020). Secondary sensitizations in the pain system are also postulated to occur, resulting in a state of hyperexcitation in the pain control system pathways (Barreto et al. 2020). It appears that a greater pull on the hair is more likely to result in an external‐traction headache. It has been documented that individuals are more likely to experience headaches when their hair is wet rather than dry (Blau 2004).

The pathophysiology of external‐traction headache is largely speculative as well.

### 4.4 Therapy

The cessation of external‐traction headaches can be achieved through the release of the hair when pulled, the loosening of the binding, or the loosening of the braid termination (Barreto et al. 2020). The application of manual massage to the affected areas, using either the fingers or the palm of the hand, was frequently cited as a method for alleviating pain (Barreto et al. 2020). Additionally, it has been reported that tying the hair 1 cm from the scalp can provide relief from external‐traction headaches. Similarly, a light scalp massage has been shown to be an effective method for alleviating external‐traction headaches (Blau 2004).

Our literature research also found no evidence that alternative therapeutic measures are effective. There is a need for further research. Table 5 summarizes practical recommendations for the prevention of external‐traction headache.

**TABLE 5:** Practical strategies based on our experience to avoid external‐traction headache.

*Avoidance of the precipitating cause*: If possible, remove the triggering factor.
*Avoid tight headgear*: If wearing headgear (like helmets, hats, or headbands), ensure they fit properly and are not too tight to prevent traction on the head.
*Reduce weight on head*: Avoid carrying heavy bags or loads that can strain the neck and lead to tension.
*Frequent breaks*: Take short breaks every 30–60 min to stand, stretch, and move around, reducing muscle tension in the neck and shoulders.
*Micro‐exercises*: Incorporate quick neck and shoulder stretches during breaks to relieve tightness.
*Relaxation practices*: Use techniques such as deep breathing, mindfulness, or meditation to lower overall stress levels and reduce muscle tension.
*Yoga or pilates*: Participate in activities that promote relaxation and flexibility, helping to relieve tension in the neck and shoulders.
*Consistent schedule*: Maintain a regular sleep schedule to ensure adequate rest and recovery.

### 4.5 Associations With Other Headache Disorders

The latency and duration of an external‐traction headache attack are not contingent on the presence of a primary headache precondition or migraine disorder. Migraine patients have a lower pain threshold, so that external‐traction headaches could occur more frequently in these people (Barreto et al. 2020). The presence of a primary headache precondition or migraine disorder is associated with an increased likelihood of developing an external‐traction headache (Barreto et al. 2020).

## 5 Summary

External‐compression headaches and external‐traction headaches lead to an impairment of quality of life if the triggering factor cannot be eliminated for cultural, religious, or safety reasons. Although both headaches are relatively common in specific communities, they are rarely represented in the scientific literature. The available literature lacks data on the general prevalence of the two types of headaches; in particular, the frequency of traction headache has not yet been sufficiently investigated in the general population. The recommendations for (non‐)medication treatment and prophylaxis are rudimentary. There is also room for improvement in the pathophysiological understanding of the two types of headaches, and it is possible that intensified research in this area would stimulate headache research as a whole. When developing specific head protection, for example, for motorcyclists or military personnel, special attention should be given to the avoidance of compression headaches. Recognition as an occupational disease should be considered.

## Author Contributions

**Ole Hensel**: conceptualization, investigation, writing–original draft, visualization, methodology, formal analysis. **Torsten Kraya**: conceptualization, investigation, methodology, formal analysis, data curation, writing–review and editing.

## Conflicts of Interest

The authors declare no conflicts of interest.

### Peer Review

The peer review history for this article is available at https://publons.com/publon/10.1002/brb3.70202.

## Data Availability

Data sharing is not applicable to this article as no new data were created or analyzed in this study.
